# Effect of Amlodipine/Nebivolol combination therapy on central BP and PWV compared to Amlodipine/Valsartan combination therapy

**DOI:** 10.1186/s43044-022-00254-0

**Published:** 2022-03-14

**Authors:** El-Zahraa M. Sultan, Hoda Rabea, Ahmed A. Elberry, Hesham B. Mahmoud

**Affiliations:** 1 Cardiology Department, Beni-Suef University Hospital, Beni-Suef City, 62511 Egypt; 2 Clinical Pharmacy Department, Faculty of Pharmacy, Beni-Suef University, Beni-Suef City, 62511 Egypt; 3 Department of Pharmacy Practice, Pharmacy Program, Batterjee Medical College, Jeddah, 21442 Saudi Arabia; 4 Beni-Suef University Hospital, Beni-Suef City, 62511 Egypt

**Keywords:** Amlodipine/Nebivolol, Amlodipine/Valsartan, Combination therapy, Hypertension, Central blood pressure, Arterial stiffness

## Abstract

**Background:**

Pulse wave velocity (PWV) and central blood pressure (CBP) have been intoduced into managment of hypertensive patients. PWV is positively correlated with arterial wall stiffness while central aortic pressure becomes better predictor of cardiovascular outcome than peripheral pressure. Reduction in CBP provides protective properties against subclinical organ damage. This work aims to investigate the effect of a new combination therapy of Amlodipine/Nebivolol (A/N) on central BP, peripheral BP and PWV. The results of using this combination will be compared to the well-established fixed-dose combination of Amlodipine/Valsartan (A/V). The study conducted between October 2018 and August 2020. One hundred and two hypertensive patients were assigned for Amlodipine 10 mg/Valsartan 160 mg combination therapy (A/V, *n* = 52) or Amlodipine 10 mg/Nebivolol 5 mg combination therapy (A/N, *n* = 50) by simple 1:1 randomization. Office, central blood pressure and PWV were measured on first (0 week), second (4–8 weeks) and third visit (10–12). Difference in BP (in each arm and between arms) was calculated along all visits.

**Results:**

No statistical significant difference was found between A/V and A/N regarding age, gender, BMI and CV history. OBP, CBP and PWV were significantly reduced in each arm, but no differences were found when comparing both arm results to each other. Recorded side effects were insignificant.

**Conclusions:**

The new combination therapy Amlodipine/Nebivolol (A/N) affords a significant reduction in CBP, PBP and PWV with minor and tolerable side effects. It has provided comparable results to Amlodipine/Valsartan (A/V) combination therapy.

## Background

Central aortic pressure (CBP) becomes a better predictor of cardiovascular outcome than peripheral pressure, particularly in individuals with prominent pulse pressure (PP) amplification [1, 2]. Reduction in CBP by some antihypertensive drugs provides protective properties against subclinical organ damage [3]. CBP was initially measured directly by using invasive devices, and then, other methods have been established to assess central pressures based on analyses of carotid and radial pulses or carotid distension waves [4]. Pulse wave velocity (PWV) may predict the cardiovascular mortality and morbidity of hypertensive patients [5]. PWV becomes a marker for arterial stiffness and is associated with atherosclerosis of the aorta. Higher arterial PWV values represent greater stiffness. Its monitoring through a noninvasive method is a useful way of evaluating the degree of atherosclerosis and predicting all-cause and cardiovascular mortality [6]. According to previous data [7, 8], some antihypertensive medications have been shown to influence arterial stiffness with different degrees of effect. This work aims to investigate the effect of a new combination of Amlodipine/Nebivolol (A/N) on central BP and PWV compared to the well-established fixed-dose combination of Amlodipine/Valsartan (A/V).

## Methods

**Fig. 1:**
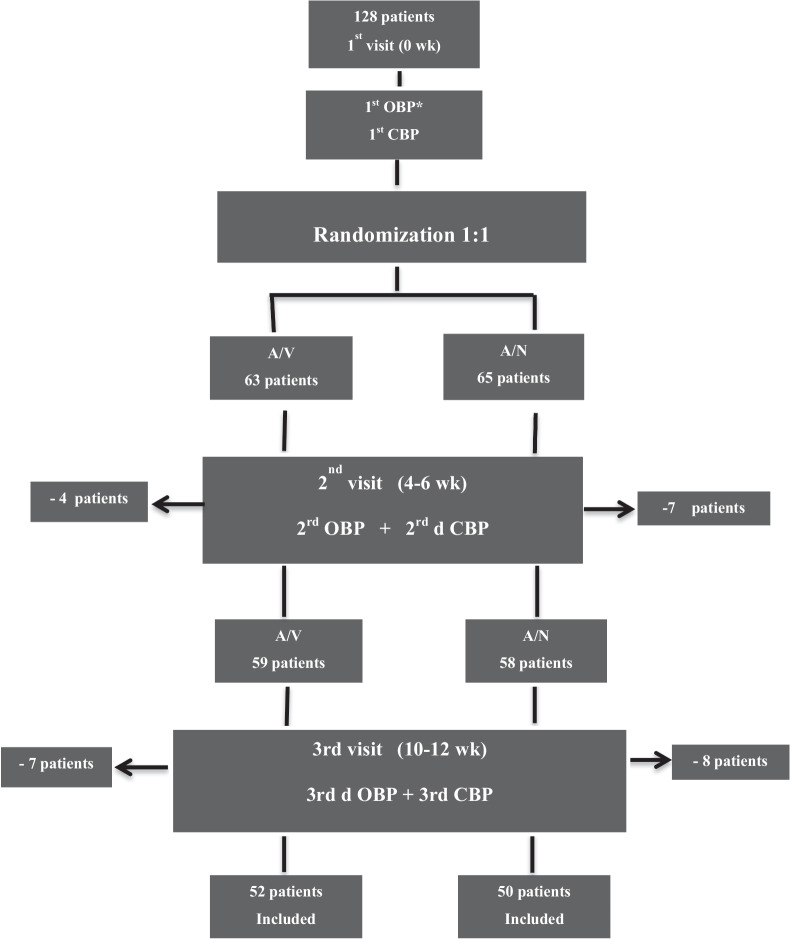
Study protocol. *Diagnosis is permitted by office BP using calibrated mercury sphygmomanometer for all patients and by Mobil-O-Graph for CBP

This is a prospective open-label randomized study which aims to investigate the effect of Amlodipine 10 mg/Nebivolol 5 mg (A/N) combination therapy in comparison with Amlodipine 10 mg/Valsartan 160 mg (A/V) on central hemodynamics parameters. The study was conducted in a hypertension clinic in hospital between October 2018 and August 2020. All moderate to severe adult hypertensive patients were included. Patients with contraindication to Amlodipine, Valsartan and Nebivolol or pregnant, lactating women or females of childbearing potential not practicing contraception were excluded. The included hypertensive patients were either newly diagnosed or previously received antihypertensive medications and still with uncontrolled hypertension (Table 1). Antihypertensive medications were substituted with either of the suggested combination through the randomization process. Patients were randomized by stratifying according to age (≥ 55 years old) and sex then by simple 1:1 randomization. One hundred and twenty-eight hypertensive patients were assigned for Amlodipine 10 mg/Valsartan 160 mg combination therapy (A/V) or Amlodipine 10 mg/Nebivolol 5 mg combination therapy (A/N). Twenty-six patients were excluded over follow-up visits (Fig. 1). Statistical analysis was applied on the final number of patients; one hundred and two patients, divided as: 52 patients in A/V group and 50 patients in A/N group. Office, central blood pressure and hemodynamics parameters were measured on first (0 weeks), second (4–6 weeks) and third visit (10–12 weeks). Difference in BP (in each group and between groups) was calculated across the three separate visits. At the third visit, ambulatory blood pressure (AMBP) was done for patients with uncontrolled blood pressure to exclude white coat hypertension (WCH). Orthostatic hypotension, rash, headache were looked for as a side effects for treatments in follow-up visits.

**Table 1:** Baseline patients’ characteristics

Variable	A/V [*n* = 52 (%)]	CI	A/N [*n* = 50 (%)]	CI	*P* value
Demographic					
Age (mean ± SD) years	57.5 ± 10.7	[54.5, 60.5]	59.4 ± 10.4	[56.4, 62.3]	0.382
Sex					
Male	18 (34.6)		19 (38.0)		0.722
Female	34 (65.4)		31 (62.0)		
BMI (mean ± SD)	31.9 ± 5.0	[30.5, 33.3]	31.7 ± 6.1	[30.0, 33.5]	0.859
CV history					
HTN					
Chronic	49 (94.2)		48 (96.0)		0.679
Naive	3 (5.8)		2 (4.0)		
DM	21 (40.4)		14 (28.0)		0.188
Dyslipidemia	29 (55.8)		34 (68.0)		0.204
Dyslip.TT					
Rosuvastatin	22 (42.3)		31 (62.0)		0.155
Atorvastatin	8 (15.4)		5 (10.0)		
Atorvastatin/EZT	2 (3.8)		0 (0.0)		
No	20 (38.5)		14 (28.0)		
AF	7 (13.5)		6 (12.0)		0.825
Smoking					
Smoker	5 (9.6)		5 (10.0)		0.996
Non-smoker	44 (84.6)		42 (84.0)		
Ex-smoker^	3 (5.8)		3 (6.0)		

A/V, Amlodipine/Valsartan; A/N, Amlodipine/Nebivolol; BMI, body mass index, CV, cardiovascular; HTN, hypertension; TT, treatment; DM, diabetes mellitus; IHD, ischemic heart disease; AF, atrial fibrillation; Dyslip, dyslipidemia; EZT, ezetimibe

^Ex-smoker; smoking cessation ≥ 6 month

*A *p*-value less than or equal 0.05 is statistically significant

Demographic data and past medical history collected. Assessment of hypertension-mediated organ damage was accomplished by 12-lead ECG, echocardiography, urine albumin-to-creatinine ratio and fundus examination. Echocardiography was performed for all patients in the left lateral position in multiple windows using PHILIPS HD11 and VIVID S5 GE machine. Ejection fraction (EF), left ventricular (LV) dimensions, septal and posterior wall thickness and valvular affection were obtained. Diastolic dysfunction was also assessed. Albumin-to-creatinine ratio was used as a marker for nephropathy assessment. LVH was detected by ECG and echocardiography.

A/V combination is a single-pill combination was administrated on fixed daily dose, day or night, while A/N combination is a two-pill combination were administrated separately on fixed times. Amlodipine 10 mg is administrated at day time while Nebivolol 5 mg is administrated at night.

Diagnosis, patient’s inclusion and follow-up visits depended on office blood pressure readings. Three office blood pressure measurements were obtained by properly calibrated mercury sphygmomanometer at each visit by the same operator. A standard operating procedure for measuring BP was followed [9–11]. For each patient, BP was measured twice at 30-s intervals. Central blood pressure was measured by the Mobil-O-Graph®—the 24-h PWA Monitor calibrated device [12]. Measurement range of this device is 60–290 mmHg for systolic and 30–195 mmHg for diastolic blood pressure, pressure range (0 to 300 mmHg) and pulse range (30–240 bpm). The device uses an oscillometric method and its accuracy level is ± 3 mmHg. The device measures the brachial systolic and diastolic blood pressure, and then, the cuff re‐inflates at the level of the diastolic blood pressure for approximately 10 s. The sensor records brachial pressure waveforms. An aortic pulse wave is then derived by using a generalized transfer function. Several pressure and cardiovascular parameters including AIx are computed by using the ARCSolver algorithm [13].

### Statistical analysis

Data were analyzed using the software, Statistical Package for Social Science (SPSS Inc. Released 2009, PASW Statistics for Windows, version 18.0: SPSS Inc., Chicago, Illinois, USA). Frequency distribution, as a percentage and descriptive statistics in the form of mean and standard deviation, was calculated. Chi-square, t-test and correlations were done in order to compare the two groups in relation to age, gender, BMI, HTN, DM, dyslipidemia, AF and smoking; *P* values of less than 0.05 were considered significant.

## Results

We tested data normality before conducting the analysis, and the data were normally distributed

### Baseline data

#### Demographic data and patients’ characteristics

Baseline patients’ characteristics were comparable between both groups. No statistical significant difference was found between A/V and A/N regarding age, gender, BMI and CV history (Table 1).

#### Echocardiographic baseline data

Echocardiographic baseline data in both groups showed no statistically significant difference (Table 2). Ejection fraction and myocardium dimension were normal in both A/V and A/N groups. Diastolic dysfunction (DD) was higher in A/N group; *P* = 0.006.

**Table 2:** Baseline investigations and target organ damage

	A/V [*n* = 52 (%)]	A/N [*n* = 50 (%)]	*P* value
Investigations			
Lipid profile			
CH	179.7 ± 43.7	171.7 ± 43.7	0.355
TG	145.3 ± 57.5	148.0 ± 57.2	0.808
LDL	106.2 ± 39.1	104.3 ± 32.5	0.786
HDL	49.6 ± 17.52	52.2 ± 22.0	0.505
AL/Cr	49.7 ± 120.3	41.6 ± 116.3	0.729
Fundus examination^			
G0	49 (94.2)	44 (88.0)	0.464
G1	2 (3.8)	2 (4.0)	
G2	1 (1.9)	2 (4.0)	
G3	0 (0.0)	2 (4.0)	
Echocardiographic data			
EF % (mean ± SD)	64.2 ± 5.9	64.1 ± 6.5	0.954
IVS	1.0 ± 0.1	1.0 ± 0.1	0.712
PWT	1.0 ± 0.4	1.1 ± 0.7	0.442
Target organ damage			
LVH	10 (19.2)	9 (18.0)	0.873
Retinopathy	3 (5.8)	6 (12.0)	0.267
Stroke	1 (1.9)	0 (0.0)	0.324
Nephropathy	7 (13.5)	5 (10.0)	0.588

A/V, Amlodipine/Valsartan; A/N, Amlodipine/Nebivolol; CH, total cholesterol; TG, triglycerides; LDL, low-density lipoprotein; HDL, high-density lipoprotein; AL/CR, albumin-to-creatinine ratio; EF, e ejection fraction; IVS, intraventricular septum; PWT, posterior wall thickness; LA, left atrium; DD, diastolic dysfunction; LVH, left ventricular hypertrophy

*A *p*-value less than or equal 0.05 is statistically significant

^Fundus examination Grades; G0: No visible abnormalities, G1: Diffuse arteriolar narrowing, G2: G 1 + focal arteriolar constriction, G3:G2 + retinal hemorrhage

#### Target organ damage baseline and laboratory investigations

No statistically significant difference was found between groups regarding target organ damage (TOD), left ventricular hypertrophy (LVH), retinopathy, stroke and nephropathy. *P* values are illustrated in Table 2. Mean values of CH, TG, LDL and HDL did not show statistically significant difference in both A/V and A/N; *P* = 0.355, *P* = 0.808, *P* = 0.786 and *P* = 0.505. Al/Cr ratio and fundus examination also did not show significant difference in both groups (Table 2).

### Baseline blood pressure

Office blood pressure (OBP) and CBP had no statistically significant difference between both groups. Mean OBP value was 158.3 ± 15.0/99.9 ± 8.4 in A/V vs. 154.7 ± 12.3/97.7 ± 6.1 in A/N (Table 3). Mean value of CBP was 135.9 ± 14.7/93.6 ± 11.4 in A/V and 135.2 ± 11.3/91.5 ± 9.5 in A/N. PBP was not statistically significant in both groups. PWV, AIX, HR and PP were not statistically different; *P* = 0.443, *P* = 0.236, *P* = 0.039 and *P* = 0.730, Table 3.

**Table 3:** Central and peripheral blood pressure at each visit

	A/V [*n* = 52 (%)]		A/N [*n* = 50 (%)]	
	Systole	Diastole	Systole	Diastole
OBP				
1st	158.3 ± 15.0 [154.1, 62.5]	99.9 ± 8.4 [97.5, 102.2]	154.7 ± 12.3 [151.2, 158.1]	97.7 ± 6.1 [95.9, 99.4]
2nd	133.9 ± 11.2	84.7 ± 7.3	134.6 ± 10.3	83.8 ± 7.8
3rd	126.1 ± 8.8	78.6 ± 6.8	123.1 ± 10.0	79.0 ± 7.8
CBP				
1st	135.9 ± 14.7 [131.8, 40.0]	93.6 ± 11.4 [90.4, 96.8]	135.2 ± 11.3 [132.0, 138.4]	91.5 ± 9.5 [88.8, 94.3]
2nd	118.9 ± 7.6	83.9 ± 10.0	121.6 ± 9.5	84.9 ± 8.1
3rd	113.4 ± 8.5	78.8 ± 9.9	114.2 ± 12.3	79.5 ± 8.2

A/V, Amlodipine/Valsartan; A/N, Amlodipine/Nebivolol; OBP, office blood pressure; CBP, central BP; 1st, first visit without medications (A/V or A/N); 2nd, second visit (on either A/V or A/N); 3rd, visit (on either A/V or A/N)

### After treatment data

#### Changes in blood pressure between visits (baseline, second and third)

i. **Office Blood Pressure (OBP)**

**Fig. 2:**
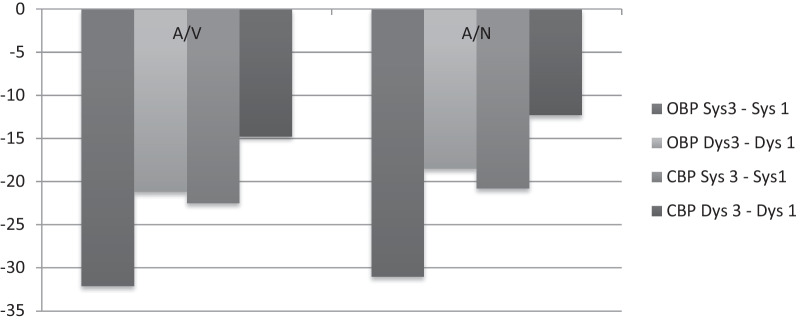
Mean differences of BP within the same group and between groups

OBP difference between all visits showed no statistically significant difference between both groups; P sys = 0.168 and P dys = 0.936, Tables 3 and 4. However, difference between (Sys2–Sys1/dys2–dys1), (Sys3–Sys2/dys3–dys2) and (Sys3–Sys1/dys3–dys1) was statistically significant within each group (Fig. 2).

ii. **Central Blood Pressure (CBP)**

**Table 4:** Difference in BP over follow-up visits

Difference in BP	AV	CI	AN	CI	*P* wave
OBP Sys3–Sys 1	− 32.1	− 37.1, − 27.2	− 31	− 34.5, − 27.4	0.168
OBP Dys3–Dys 1	− 21.2	− 24.2, − 18.2	− 18.5	− 21.2, − 15.8	0.936
CBP Sys 3–Sys1	− 22.5	− 27.1, − 17.8	− 20.8	− 25.2, − 16.5	0.331
CBP Dys 3–Dys 1	− 14.8	− 18.3, − 11.2	− 12.3	− 15.5, − 9.1	0.318

A/V, Amlodipine/Valsartan; A/N, Amlodipine/Nebivolol; OBP, office blood pressure; CBP, central BP; 1st, first visit without medications (A/V or A/N)

CBP difference between all visits did not reveal statistically significant difference between A/V and A/N group; P sys = 0.331and P dys = 0.318. For each group, reduction in CBP in every visit was statistically significant compared to the previous one (Fig. 2, Table 4).

**Fig. 3:**
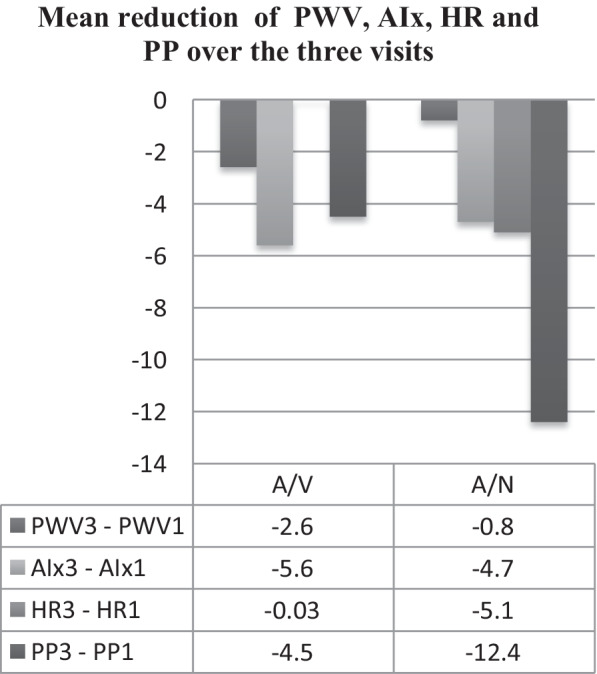
Mean differences of central hemodynamics within the same group and between groups

Reduction in HR was statistically significant only in A/N group; *P* value of HR 3–HR 1 = 0.017. No statistical difference was found regarding PP between both groups; *P* = 0.155 (Fig. 3).

#### Changes in central hemodynamics between visits (baseline, second and third)

Difference in PWV was not significant between groups; *P* = 0.379, while difference within A/N group was statistically significant (*P* value of PWV 2–PWV 1 = 0.000), (*P* value of PWV 3–PWV 2 = 0.003) and (*P* value of PWV 3–PWV 1 = 0.000) (Fig. 3). AIX also did not show a significant difference between groups; *P* = 0.803. AIX difference within A/N group (AIX 3–AIX 2) and (AIX 3–AIX 1) was statistically significant; *P* = 0.003 and *P* = 0.000 (Fig. 3).

### Safety monitoring during treatment

In A/N, 6 patients had lower limb edema. One patient had headache and no cases of rash were reported. Eight patients in A/V had lower limb edema; two patients had headache and no rash cases were reported.

### Excluded patients during the study

Across the three follow-up visits, 15 patients were excluded from A/N group and 11 from A/V group, due to lack of patient compliance to treatment or because they had already started taking other medications such as NSAID or alpha-agonist drugs.

## Discussion

To our knowledge, this is the first study to address the safety and efficacy of Amlodipine/Nebivolol (A/N) combination itself or to compare it with the well-established combination of Amlodipine/Valsartan (A/V). BP reduction in hypertensive patients is associated with a lower rate of cardiovascular events[12]. Combination of A/N showed significant reduction of office and central BP through all visits (*P* = 0.000) (Table 3). Central BP reduction was accompanied with decrease in PWV over the second and third visit. This result is attributed to the combined vasodilatory effect of Amlodipine with Nebivolol through releasing nitric oxide and improving the endothelial function, which reduces arterial stiffness (AS) [8]. Long-term use of Nebivolol showed promising results regarding CBP and AS compared to other b-blockers [13–17]. Rather than Nebivolol, other types of B-blocker were inferior compared to ARBs [18]. Significant effect of Amlodipine on brachial and central BP had been investigated in previous studies. Improvement of AIX was notable [16, 19, 20]. Amlodipine effect in A/V combination therapy on central BP and PWV has been established before [21, 22]. Patients in A/V group in the current study had results, which agree with previously published data. Significant reduction in brachial and central BP was obtained within A/V group, while patients in A/N group had comparable results to those in A/V group regarding OBP, CBP and PWV (Figs. 2, 3). CBP difference (Sys3-1/Dys3-1) in A/V was − 22.5 ± 16.6/− 14.8 ± 12.8 mmHg vs. − 20.8 ± 15.0/− 12.3 ± 11.0 mmHg in A/N group.

Target OBP (< 140/90) was not obtained in four patients in A/N group versus five patients in A/V group (140/80, 140/80, 140/85 and 150/100 vs. 140/80, 140/85, 140/85, 150/80 and 160/90). Ambulatory blood pressure (AMBP) was done for those patients to exclude white coat hypertension (WCH); this revealed two patients with white coat hypertension in A/N versus one patient in A/V group. Patients with WCH in the A/N group had uncontrolled AMBP during night times only. This result could be attributed to the morning administration of Amlodipine instead of night administration. Flack et al. [23] had discussed the effect of single daily antihypertensive drug in comparison with twice daily administration on blood pressure variability. This study concluded that long acting drugs provided high trough-to-peak ratio which allowed better BP control during the nighttime and early morning hours. This eliminates the need to use high dose of short acting drugs to obtain high trough-to-peak ratio; this will lead to fewer adverse events. The timing of the drug administration also can affect the early morning surge in BP which eventually decreases the blood pressure variability. Theoretically, A/N could have intermediate to high trough-to-peak ratio; however, Amlodipine has longer duration of action than Nebivolol. Night administration of Amlodipine would be contributed to better coverage until early morning.

PWV values are affected by aging process [24, 25], impaired Glucose level [26], obesity [27] and smoking [28, 29], but not by gender which agrees with previously published studies. Both A/V and A/N revealed no difference in baseline age or gender (*P* = 0.382 and *P* = 0.722). Diabetes, dyslipidemia, BMI and smoking status are comparable in both groups. Patients on A/N combination had significant reduction in PWV; this was revealed through the three follow-up visits (*P* = 0.000) with no final statistical difference in comparison with those on A/V (*P* = 0.379).

Using AIx as a co-marker for AS [30, 31]) in hypertensive patients could provide more knowledge about patient vascular aging in status versus the real biological aging. In our study, AIx decreased over the second and the third visit in each group with no statistical difference between both groups (*P* = 0.803).

Reduction in HR was significant only within A/N patients (*P* = 0.017), but not between both groups (*P* = 0.155). Baseline HR was not statistically different between both groups; A/V HR = 80.8 ± 14.5 and A/N HR = 74.9 ± 14.2 (*P* = 0.039).

Increased PP is a significant risk factor for development of heart disease and vital organ damage especially the brain and the kidneys; a 10 mmHg increase in the pulse pressure raises the cardiovascular risk by as much as 20% [32]. Wide PP (> 100 mmHg) is associated with extensive cardiovascular disease and is an independent predictor of disease progression and all-cause mortality [33–35]. Values of baseline PP in both groups A/V and A/N were not wide (53.7 ± 191.1 and 55.2 ± 18.0; *P* = 0.730). PP values were decreased significantly within A/N over the three follow-up visits, but not in A/V group (*P* = 0.002) vs. (*P* = 0.234) (Fig. 3).

A/N combination had a low rate of minor side effects such as lower limb edema and headache as in A/V group. Orthostatic hypotension was reported in three elderly patients in A/N group at the second week after starting the treatment and disappeared in the following visits. This could be attributed to the combined vasodilatory effect of Nebivolol and Amlodipine.

## Future directs

A/N combination would be investigated in hypertensive patients with ischemic heart disease or heart failure with preserved ejection fraction.

## Limitations

Since A/N is a newly proposed fixed-dose combination therapy which is not available in the form of a single-pill combination, Nebivolol 5 mg and Amlodipine 10 mg were administrated as two pills. The administration time of both pills is fixed through the day, but it may be affected by patient commitment and compliance at home, which may impact the timing of the two drugs release throughout the day. Finally, the single-center trial was another limitation; larger sample size and multicenter trials are needed to provide more comprehensive results.

## Conclusions

The new combination therapy Amlodipine/Nebivolol (A/N) affords a significant reduction in CBP, PBP and PWV with minor and tolerable side effects. It has provided comparable results to the well-known Amlodipine/Valsartan (A/V) combination therapy.

## Data Availability

Available upon request.

## Abbreviations

AMBP: Ambulatory blood pressure
A/V: Amlodipine 10 mg/Valsartan 160 mg
A/N: Amlodipine 10 mg/Nebivolol 5 mg
AS: Arterial stiffness
AIX: Augmentation index
CBP: Central aortic pressure
DD: Diastolic dysfunction
EF: Ejection fraction
HR: Heart rate
HDL: High-density lipoprotein
LV: Left ventricular
LVH: Left ventricular hypertrophy
LDL: Low-density lipoprotein
OBP: Office blood pressure
PP: Pulse pressure
PWV: Pulse wave velocity
TOD: Target organ damage
CH: Total cholesterol
TG: Triglycerides
WCH: White coat hypertension
